# Correction: Speed variations of bacterial replisomes

**DOI:** 10.7554/eLife.88685

**Published:** 2023-04-19

**Authors:** Deepak Bhat, Samuel Hauf, Charles Plessy, Yohei Yokobayashi, Simone Pigolotti

**Keywords:** *E. coli*

Bhat D, Hauf S, Plessy C, Yokobayashi Y, Pigolotti S. 2022. Speed variations of bacterial replisomes. *eLife* **11**:e75884. doi: 10.7554/eLife.75884.

Published 25 July 2022

We noticed a few minor errors after publication.

The first error is related with Eq. (9) of the paper. In deriving Eq. (9), we assumed that the dynamics of the two replisomes is symmetric, so that $p^{st}\left(x_{1},x_{2}\right)=p^{st}\left(x_{2},x_{1}\right)$ . This assumption holds for all the models considered in the paper. However, we have not explicitly stated that Eq. (9) is derived under this assumption. Moreover, we realized that one can not identify each of the two integrals in Eq. (9) with the contribution coming from one of the two replisomes.

Corrected equation and following sentence:

(9) $$P(y)=\overset{y\;in\;an\;incomplete\;genome}{\overbrace{\frac{k}{\beta +k}[\int_{|y|}^{L}dx_{1}\int_{0}^{L-x_{1}}dx_{2}\;p^{st}(x_{1},x_{2})+\int_{L-|y|}^{L}dx_{2}\int_{0}^{L-x_{2}}dx_{1}\;p^{st}(x_{1},x_{2})}}]+\overset{y\;in\;a\;complete\;genome}{\overbrace{\frac{\beta }{k+\beta }}}\;,$$

where we assumed that the dynamics of the two replisomes is symmetric, so that $p^{st}\left(x_{1},x_{2}\right)=p^{st}\left(x_{2},x_{1}\right)$, and we used that a randomly chosen genome is complete with probability $\left(1-N_{S}/N)=\beta /(k+\beta \right)$, see Equation 5.

Original equation and following text:

(9) $$P(y)=\frac{k}{\beta +k}[\overset{y\;copied\;by\;1^{st}\;replisome}{\overbrace{\int_{|y|}^{L}dx_{1}\int_{0}^{L-x_{1}}dx_{2}\;p^{st}(x_{1},x_{2})}}+\overset{y\;copied\;by\;2^{nd}\;replisome}{\overbrace{\int_{L-|y|}^{L}dx_{2}\int_{0}^{L-x_{2}}dx_{1}\;p^{st}(x_{1},x_{2})}}]+\overset{y\;in\;a\;complete\;genome}{\overbrace{\frac{\beta }{k+\beta }}}\;.$$

where the two terms in square brackets reflect the fact that either of the two replisomes can in principle have copied position y, and we used that a randomly chosen genome is complete with probability $\left(1-N_{S}/N)=\beta /(k+\beta \right)$, see Equation 5.

The second error regards the sentence in the discussion: “Similarly, oscillations in the mutation rate are also less evident at lower temperature and disappear in minimal medium (Niccum et al. 2019).” The first part of this statement is incorrect: Niccum et al. 2019 did observe evident oscillations at low temperature. We remark that their observation is not in disagreement with our hypothesis that the variations in replication speed and mutation rate stem from a common cause. In fact, the lowest temperature studied in (Niccum et al. 2019) is T=22 C. At this temperature, we observe pronounced oscillations in replisome speed as well (see Figure 3 in the eLife paper).

Removed sentence:

Similarly, oscillations in the mutation rate are also less evident at lower temperature and disappear in minimal medium (Niccum et al. 2019).

The third correction is related with the library preparation for sequencing. In the “Sequencing” subsection of the Methods section, the name of the kit used for library preparation was not accurate.

Corrected sentence:

Libraries were then prepared using the PCR-free NEBNext Ultra II DNA Library Prep Kit for Illumina.

Original sentence:

Libraries were prepared using the Illumina DNA PCR-Free Library Prep Kit.

The article has been corrected accordingly.

